# Validation of Quantitative HPLC Method for Bacosides in KeenMind

**DOI:** 10.1155/2015/696172

**Published:** 2015-08-27

**Authors:** Ashley Dowell, George Davidson, Dilip Ghosh

**Affiliations:** ^1^Southern Cross Plant Science, Southern Cross University, Lismore, NSW 2480, Australia; ^2^Soho Flordis International Pty Ltd., 156 Pacific Highway, St. Leonards, NSW 2065, Australia

## Abstract

Brahmi (*Bacopa monnieri*) has been used by Ayurvedic medical practitioners in India for almost 3000 years. The pharmacological properties of* Bacopa monnieri* were studied extensively and the activities were attributed mainly due to the presence of characteristic saponins called “bacosides.” Bacosides are complex mixture of structurally closely related compounds, glycosides of either jujubogenin or pseudojujubogenin. The popularity of herbal medicines and increasing clinical evidence to support associated health claims require standardisation of the phytochemical actives contained in these products. However, unlike allopathic medicines which typically contain a single active compound, herbal medicines are typically complex mixtures of various phytochemicals. The assay for bacosides in the British Pharmacopoeia monograph for* Bacopa monnieri *exemplifies that only a subset of bacosides present are included in the calculation of total bacosides. These results in calculated bacoside values are significantly lower than those attained for the same material using more inclusive techniques such as UV spectroscopy. This study illustrates some of the problems encountered when applying chemical analysis for standardisation of herbal medicines, particularly in relation to the new method development and validation of bacosides from KeenMind.

## 1. Introduction

The human brain is a complex organ that neuroscientists are still attempting to understand. As people live longer, dysfunction of the brain is becoming a predominant issue for the healthcare system. Cognitive decline, particularly in elderly people, often derives from the interaction between age-related changes and age-related diseases and covers a wide spectrum of clinical manifestations, from intact cognition through mild cognitive impairment and dementia. Neural dysfunction of the brain is becoming a predominant issue for the healthcare system as a result of human longevity.

In recent years, the interest in the use of herbal products has grown exponentially, particularly in the western world as well as in developed countries [1]. It is now becoming exceedingly apparent that available psychotherapeutics does not properly meet therapeutic demands of a vast majority of patients with mental health problems and that herbal remedies remain to be the alternative therapeutic hope for many such patients. In the folklore of Indian medicine, several herbs have been used traditionally as brain or nerve tonics. One of the most popular of these herbs is* Bacopa monniera* Wettst. (syn.* Herpestis monniera*), which belongs to the family Scrophulariaceae.

## 2. Materials and Methods

### 2.1. Brief Description of the Plant

Brahmi [2–6] has been used by Ayurvedic medical practitioners in India for almost 3000 years. The earliest chronicled mention is in the Ayurvedic treatise, the Charaka Samhita (100 A.D.), in which Brahmi is recommended in formulations for the management of a range of mental conditions including anxiety, poor cognition, and lack of concentration. According to the Charaka, Brahmi acts as an effective brain tonic that boosts one's capabilities to think and reason. The Sushruta Samhita [7] (200 A.D.) attributes the plant with efficacy in maintaining acuity of intellect and memory.

The herb is from a family Scrophulariaceae and is a small creeping herb with numerous branches, small oblong leaves, and light purple or small and white flowers, with four or five petals. It is found in wetlands throughout the Indian subcontinent in damp and marshy or sandy areas near streams in tropical regions. The genus* Bacopa* includes over 100 species of aquatic herbs distributed throughout the warmer regions of the world, apart from India, Nepal, Sri Lanka, China, Taiwan, and Vietnam and is also found in Florida and other southern states of the USA [8].

### 2.2. Active Constituents

Compounds responsible for the pharmacological effects of Bacopa include alkaloids, saponins, and sterols. Detailed investigations first reported the isolation of the alkaloid “brahmine” from Bacopa [9, 10]. Later, other alkaloids like nicotine and herpestine and isolation of D-mannitol and saponin, hersaponin, and potassium salts have also been reported [9, 10]. The major chemical entity shown to be responsible for neuropharmacological effects and the nootropic action or antiamnestic effect of Bacopa is bacoside.

### 2.3. Bacosides Description

The term bacosides refers to dammarane-type triterpenoid glycosides found in extracts of* Bacopa monnieri*. There are over 30 bacosides reported, with most being either jujubogenin or pseudojujubogenin glycosides.

Triterpenoid glycosides fall into the broader category of “saponins,” as their amphoteric nature allows them to form emulsions in water. Triterpenoids are widely reported actives in plant based medicines and synthetic analogues have been developed for specific pharmacological functions.

**Figure 1:**
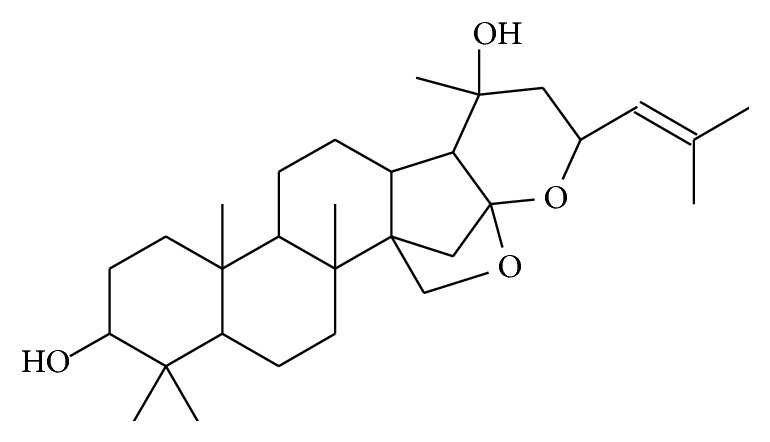
Jujubogenin MW:472.707.

Bacosides were first reported by Chatterji et al. in 1963 [11] and described as Bacoside A and Bacoside B. They were isolated by crystallisation and separated by silica column chromatography and therefore categorised as only two distinct molecules. Later research demonstrated that Bacosides A and B were in fact groupings of coeluting compounds known as Bacosides A and Bacosides B, consisting of at least 4 different but closely related jujubogenin and pseudojujubogenin glycosides. Beyond those major bacosides regarded as Bacosides A and B, there are more highly glycosylated bacosides, various minor jujubogenin/pseudojujubogenin glycosides as well as cucurbitacin glycosides, and aglycone forms of both pseudojujubogenin and jujubogenin (Figure 1).

### 2.4. Analytical Techniques for Measuring Bacosides

Early methods for quantification of Bacopa saponins involved conversion to ebelin lactones by acidic hydrolysis and then measuring these by UV-spectrophotometry Pal and Sarin (1992) [12]. Bacosides, like most triterpenoids, are largely saturated and therefore have only a small UV absorbance coefficient. Ebelin lactones which can be formed by acidic hydrolysis of various triterpenoids including bacosides have a strong chromophore and are readily detected by UV-spectroscopy at 278 nm.

It was not until 2004 that Ganzera et al. [13] published the first analytical procedure to separate and quantify bacosides by HPLC. The following year, Deepak et al. [14] published a method for quantitative determination of the major saponin mixture Bacoside A in* Bacopa monnieri* by HPLC. Murthy et al. [15] followed with a similar approach to Deepak but more comprehensive in its inclusion of 12 bacosides calculated. In 2011 both the British Pharmacopoeia (BP) and the United States Pharmacopoeia (USP) for the first time included monographs for* Bacopa monnieri*. Each included an assay for bacosides by HPLC, which appear to be based upon methods described by Murthy et al. [15] and Deepak [14] respectively.

It is generally expected that compendia methods are validated and can be applied directly without requirement for further validation. While this may be workable for uncomplex pharmaceuticals, it is less realistic when applying methods to complex herbal formulas such as those made from* Bacopa monnieri*. We challenged the current BP method for quantification of a bacosides to routine method validation to assess the suitability of this method for stability evaluation of the potency of KeenMind (http://www.keenmind.info/).

Validation of analytical methods involves examining the uncertainty associated with each component of a methodological procedure as a means to assessing the suitability of a method for its desired purpose.

General procedures and parameters for validation of analytical methods for the measurement of pharmacologically active substances are guided by regulatory guidelines established by the WHO and PIC/S as well as National Pharmaceutical Compendia such as the BP and the USP.

**Figure 2:**
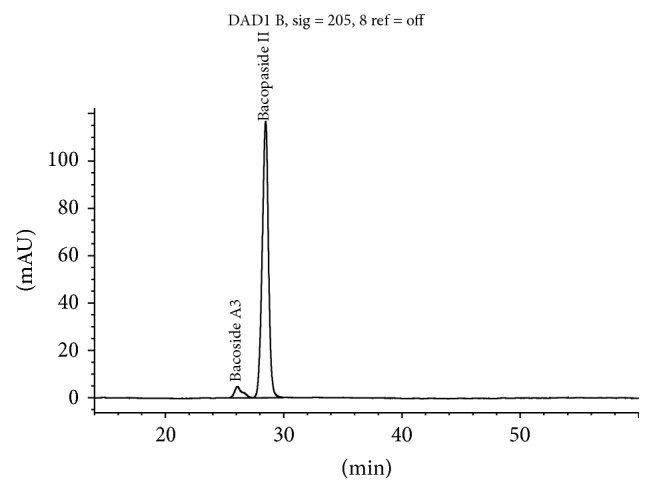
Bacopaside II HPLC by BP method at 205 nm.

The method separates bacosides by HPLC using an isocratic mobile phase with detection by UV-Vis detector at 205 nm (Figure 2). Bacopaside II is used as the calibrating standard and selected bacoside peaks are identified by their relative retention time to Bacopaside II.

We used an Agilent 1100 HPLC with a UV-Vis detector, and a reverse phase Phenomenex Synergi 250 mm × 4.6 mm HPLC column with 5 *μ*m, C18 (octadecyl) packing. The isocratic mobile phase was prepared by mixing 315 volumes of acetonitrile and 685 volumes of 0.72% w/v anhydrous sodium sulphate, previously adjusted to pH 2.3 with sulphuric acid. This was run isocratically over 75 minutes with a flow rate of 1.0 mL/min and an injection volume of 20 *µ*L.

A stock solution of the calibrating reference standard Bacopaside II was prepared diluting 5 mg into 5 mL with methanol (1 mg/mL). This was further serial diluted to create 5-point standard curve across a concentration range of approximately 1.0 to 0.01 mg/mL. The contents of 20 capsules of KeenMind were combined to provide a representative sample. Approximately one gram of the powdered extract was diluted in 70% methanol in 50 mLs and sonicated for 30 minutes, followed by centrifugation. The solution was sampled for injection onto HPLC (Tables 1 [Table tab2] [Table tab3]–4).

**Figure 3:**
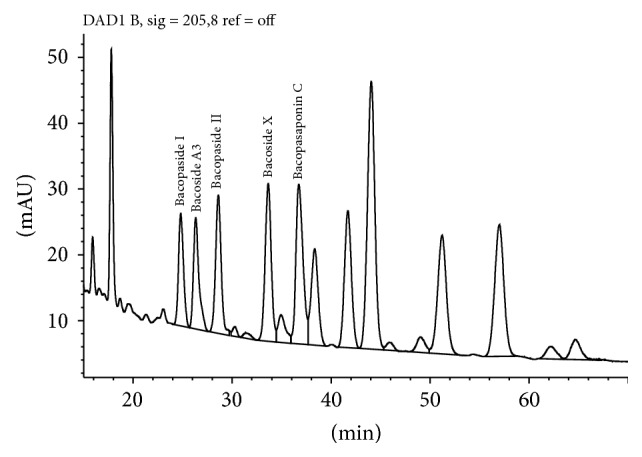
Bacoside peaks (labelled) included in total Bacosides by BP.

According to the BP, when the chromatograms are recorded using, the prescribed conditions the retention time of Bacopaside II is about 36 minutes. According to the BP, the retention times relative to Bacopaside II are as follows: luteolin, about 0.3; Bacoside A3, about 0.9; Bacoside A, about 1.2; Bacopasaponin C, about 1.3; Bacopaside I, about 1.4 (Figure 3, Table 5). According to the BP method the test is not valid unless, in the chromatogram obtained with the test solution, the resolution factor between the peaks due to Bacoside A3 and Bacopaside II is at least 1.5 and the resolution factor between the peaks due to Bacoside A and Bacopasaponin C is at least 2.4. The total content of Bacopa saponins, expressed as Bacopaside II, is calculated from the chromatograms obtained and using the declared content of Bacopaside II in the certified reference standard.

## 3. Results

### 3.1. Validation of the BP Bacosides Assay

We applied validation procedures to the current BP assay for bacosides in KeenMind* Bacopa monnieri* extract.

Validation parameters examined included specificity, linearity, limit of detection, limit of quantitation, system precision, method precision, extraction efficiency, intermediate precision, and robustness (Tables 1–6).

“Specificity” determines that the analyte/s are correctly identified and suitably distinct to allow for accurate measurement. Specificity was assessed initially using the peak purity function on HP Chemstation and by examination of peak profiles and symmetry. Because of the structural similarity of bacosides with respect to their chromophore, the peak purity function was unable to differentiate between different overlapping bacoside peaks. It did however indicate that bacoside analytes were not coeluted with compounds of a different structural class.  Table 1 shows peak purity values for the bacoside analytes calculated by BP bacosides assay. Close examination of peaks included as bacosides by the BP shows minor peaks occurring on the front tails indicating nonspecificity. Given their equivalent UV-Vis response it is likely that these represent minor bacosides.

We purchased available reference standards for the major bacosides to confirm the correctness of the BP peak identification guide which ascribes relative retention times for peaks to be calculated as bacosides relative to the retention time of Bacopaside II. According to this guide Bacopaside I is the large peak eluting at 38.5 min whilst the reference standard purchased from Sigma-Aldrich coelutes with the peak at 22.5 min (Figure 3). Further, the BP method refers to Bacoside A as being a single peak at 1.2 times the retention time of Bacopaside II. The identification of Bacoside A as a single compound is an historical error. The peak at 1.2 times the retention time of Bacopaside II is Bacopaside X, as confirmed by comparison to the purchased reference standard.

Interestingly, this method does not include bacosides eluting after 29.5 min, which are clearly evident in the trace. This effectively diminishes the value for calculated bacosides in KeenMind from about 30% to about 10%. However, clinical trials which have provided supporting evidence for claims for* Bacopa* efficacy and thereby effective dosage ranges have been standardised for total bacoside by more inclusive methods based on UV-spectroscopy. Such methods calculate total bacoside values of about 50%. This creates problems for sponsors when displaying appropriate dosage recommendations on packaging.

**Figure 4:**
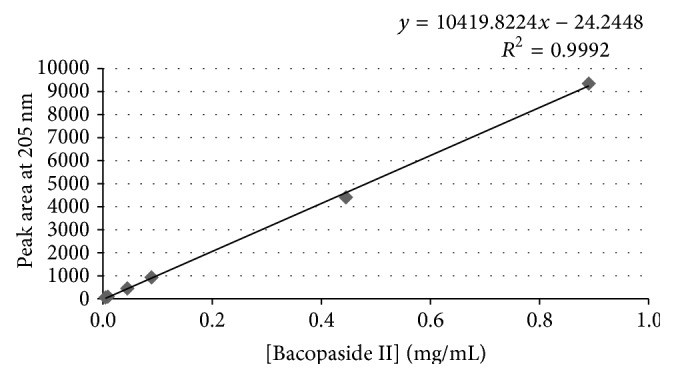
Bacopaside II calibration curve.

To assess linearity, a 6-point set of Bacopaside II, standard solutions prepared in the range of LOQ 150% of the nominal concentration were injected onto HPLC in triplicate. The linearity curve was plotted and the *R* ^2^ and *Y*-intercept were calculated. Across this range an *R* ^2^ of 0.9992 was attained where the acceptance criteria are ≥0.990 (Figure 4).

The range of concentration over which the assay is valid is determined by confirming the linear correlation of analyte concentration to instrument response; the limit of detection (LOD); limit of quantitation (LOQ); system and method precision; and accuracy/recovery. We found that the range over which precision, accuracy, and linearity met their defined criteria was from 0.0089–0.89 mg/mL.

LOD (limit of detection) is the concentration at which the analyte is detectable, but where interference from background noise occupies at least 30% (signal : noise ratio = 3) of the peak height, rendering measurement too inaccurate to be recorded. LOQ is the concentration at which the background noise occupies up to 10% (signal : noise ratio = 10) of the peak height, allowing for a reasonable estimate of peak area to be measured.

A series of standard (Bacopaside II) solutions with concentrations ranging from 0.01% of the nominal concentration of the active peak to 100% working strength of the impurities was accurately prepared. Triplicate injections of each were performed and the signal-to-noise ratios determined for all samples starting with the least concentrated. The acceptance criteria for LOD is typically S/N of all 3 injections per solution ≥3.0, which for Bacopaside II is a concentration of 0.00445 mg/mL.

For LOQ, a series of standard solutions was accurately prepared from the LOD concentration to 100% of the standard working concentration. Triplicate injections of each of the above solutions were performed and signal-to-noise ratios of all the solutions determined. The acceptance criteria for LOQ is typically S/N of all 3 injections per solution ≥10.0, which for Bacopaside II is 0.0089 mg/mL.

Range can also be limited by recovery of the analyte from the matrix in which it is bound, prior to extraction for analysis. “Recovery,” also termed “accuracy,” is typically assessed by spiking known amounts of the analyte substance into a formulation or carrier matrix and compared to the amount measured in the samples upon analysis. In our validation of the BP method we applied accuracy by both spiked addition and extraction efficiency studies.

For accuracy by spiked addition, Bacopaside II was added at known concentrations to the product placebo. 2.2 mg Bacopaside II reference standard was accurately weighed into a 10 mL volumetric flask and dissolved and diluted to volume with the solvent mix. 1 mL of this solution was accurately transferred to HPLC sample vials containing 16, 20, and 24 mg placebo and sonicated for 15 minutes. The spiked samples were analysed by HPLC and the amount of Bacopaside II calculated. The % recovery of Bacopaside II from theoretical amounts added was determined and used to indicate the impact on matrix binding of the analyte.

The acceptance criteria for accuracy are that the recovery of Bacopaside II is 90.0% to 110.0%, from concentrations ranging from 80 to 120% of nominal stated content of the analyte.  Table 3 shows accuracy data within the acceptance criteria at all three spiked addition concentrations.

In our validation of the BP bacoside assay we also applied a technique coined “extraction efficiency” wherein the test sample is extracted in series up to 5 times and each serial extract is analysed quantitatively. The total analyte from all serial extracts is determined and the respective % yield of each progressive extract calculated. This approach is used by our laboratory for herbal substances where the plant extract matrix cannot be readily replicated. It measures the efficiency of the extraction process of the analytical method.

To assess extraction efficiency, about 1 g of the sample powder was weighed into a 40 mL vial. 30 mL of methanol/water (70/30) was added and sonicated for 15 minutes. The solution was centrifuged and the supernatant transferred to a 50 mL volumetric flask and made up to mark. Another 15 mL of methanol/water (70/30) was added to the 40 mL vial containing the residual pellet, sonicated for 15 minutes and the centrifugation step repeated. The process was repeated once more and aliquots were sampled from each of the three 50 mL volumetric flasks for injection onto HPLC.

A nominal acceptance criteria of >95% were set for recovery from first extract, which is prepared according to the BP bacoside method.  Table 4 shows extraction efficiency results. Because a small volume of solvent remains in the undissolved tablet material after the supernatant has been decanted from the first extraction, the remaining analyte in this volume is then dissolved in the second extraction. As such, a small amount, typically less than 5%, will be present in the second extract. If analyte is still present in the third extract this is a clear indication that not all analyte was recovered in the first extraction.

System precision is a measure of the uncertainty associated with the instrument operation and is commonly an outcome of sample injection error. For HPLC systems, precision is assessed by measuring the % RSD of 3–6 repeat injections of the same sample, typically a reference standard dilution. For a HPLC in good operating condition acceptance criteria for the retention time are ≤1.0%, and the % RSD of the peak area is ≤10.0%. The average peak area of bacoside peaks is about 1000 mAU which equates to Bacopaside II at 0.089 mg/mL. At this concentration the instrument attained system precision for injection of Bacopaside II of 0.24% RSD for retention time of 27.889 min and 0.73% RSD for an average peak area of 934.95 mAU.

Method precision, also called repeatability, is a measure of the inherent error in sample preparation. A test sample is prepared 6 times and each preparation injected 3 times. The means of repeat injections of each sample are compared and the % RSD measured.

Table 6 shows method precision results for KeenMind when prepared according to BP bacosides. The acceptance criteria were defined such that the mean result at method working strength is within a specified range ≤5.0% RSD.

Intermediate precision is the same task performed by a second operator. A comparison of method precision with intermediate precision is an indication of the human error associated with the sample preparation methodology. This is important as a high level of skill may mask a cumbersome or problematic method.

We attained an intermediate precision result of 2.45% RSD which is comparable to that attained by the first operator. In preparation of herbal specimen for analysis, error can occur with the use of the measuring apparatus, through insufficient extraction of analytes from the product matrix as well as variable peak area calculation affected by poor resolution from other peaks absorbing in the same region at the similar retention time.

Robustness assesses the effect of minor changes to HPLC conditions on the analyte measurement. The robustness of the BP bacoside assay was examined at increased (1.1 mL/min) and decreased (0.9 mL/min) HPLC flow rates, at modified mobile phase buffer concentration (0.7, 0.71, and 0.72% Na_2_SO_4_), and also at column temperature of 29°C and 31°C compared with a control at flow rate of 1.0 mL/min and 30°C.

The chromatographic separation of bacosides was negatively affected by all system changes applied.

**Figure 5:**
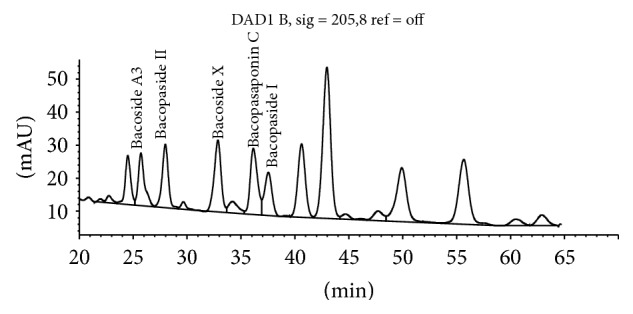
[Na_2_SO_4_] = 0.70%.

**Figure 6 fig6:**
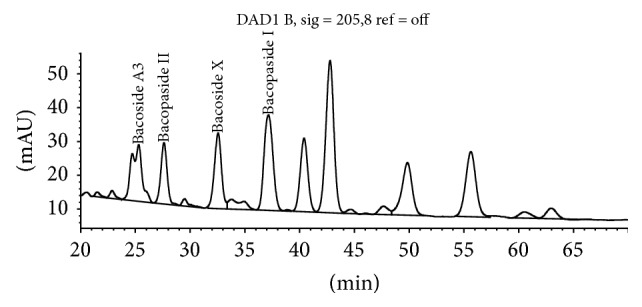
[Na_2_SO_4_] = 0.71% showing Bacopasaponin C merged with Bacopaside I.

**Figure 7:**
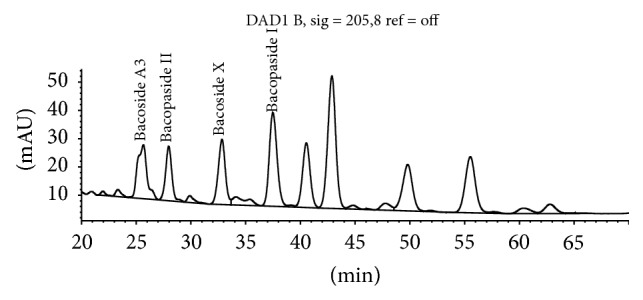
[Na_2_SO_4_] = 0.72% showing Bacoside A3 merged with peak 1.

Figures 5 [Fig fig6]–7 demonstrate the effect of adjusting the concentration of sodium sulphate in the mobile phase. These minor variations in mobile phase concentration illustrate how peaks can shift and how merged peaks can result in erroneous identification of individual bacosides.

### 3.2. Updated Clinical Efficacy of KeenMind

After twelve years of research at Swinburne University, Melbourne, KeenMind suggests that this clinically proven* Bacopa monnieri* product is a safe and efficacious cognitive enhancer [16, 17]. Robust evidence for its chronic enhancing effect is strongest, with recent studies also suggesting an acute cognitive enhancing effect [18, 19]. Additional trials with longer administration durations [20] are ongoing at Swinburne University.

## 4. Discussion and Conclusion

We found that the BP Bacopa assay is valid for the analysis of Bacopaside II as the validation results have met the acceptance criteria for this molecule (Table 7). However, the assay was not valid for accurate quantification of total bacosides due to issues with specificity as well as poor robustness and reproducibility.

Even very minor changes to HPLC conditions resulted in large shifts in peak shape and resolution. We found that the quality of separation attained was dependent upon the column condition at the time of use. This creates significant problems in stability trial evaluation where testing time-points are often months apart in which time changes to HPLC and HPLC column condition is inevitable. In order to provide reproducible stability results we run a characterised specimen of KeenMind which is stored frozen and adjust the buffer concentration of the HPLC mobile phase until optimal separation of bacosides is achieved. It is necessary to equilibrate the HPLC column in buffer solution for up to 2 hours before use and condition the column after use with blank injections of butanol. Two blank runs use a butanol injection of 20 *µ*L at the beginning of the run to confirm the absence of contaminants/carryover. To ensure the suitability of the column condition for adequate separation of bacosides a secondary reference standard is run to confirm that the peak order of elution and resolution is consistent with that prescribed by the method.

Standardisation of herbal medicines is fraught with challenges. While claiming pharmacological efficacy and having clinical evidence to support such claims, the actual mechanisms of activity are often not well understood. While we are confident that bacosides are the active constituents of* Bacopa monnieri*, we do not yet understand how these are metabolised and in what form they are functionally active. There are suggestions that Bacosides A are active while Bacosides B are not. While this may be true, differentiating between the two classes creates significant analytical problems.

Bacosides are saponins and as such have “detergent” like properties. They are therefore more susceptible to subtle change in the solid phase condition of HPLC columns. Without a buffered HPLC system, bacosides do not separate well. But this does not mean they cannot be effectively measured. Ganzera et al. [13] described a method for basic separation of bacosides from the flavonoids and phenolic acids also present in Bacopa extracts. While they did not achieve baseline separation the method is very inclusive of bacosides and produces results which are similar to those attained by the UV-spectrophotometry methods used to standardise products upon which current clinical evidence is based.

Ultimately the most accurate measure of bacosides will be achieved by gravimetric isolation using liquid/liquid partitioning and preparative column chromatography. This approach is more of research activity than being routine analytical so it is not suitable for quality control laboratories. The original ebelin lactone methods by UV-spectrophotometry are simple to apply and could be standardised by comparison to gravimetric results.

## Figures and Tables

**Table 1:** Peak purity values for BP bacoside analytes. Purity is indexed from 0 to 1000.

Compound	Peak purity
Bacoside A3	973.8
Bacopaside II	951.1
Bacoside A	827.9
Bacopasaponin C	977.8
Bacopaside I	787.5
Other peaks	>800

**Table 2 tab2:** % RSD of standard replicate injections across the calibration range.

	Concentration (mg/mL)	Mean	Standard deviation	% RSD
LOD	4.45*E* − 03	62	6.81	12.53

LOQ	8.90*E* − 03	94.84	9.47	9.98
	4.45*E* − 02	454.86	11.42	2.51

Working level	8.90*E* − 02	934.95	6.78	0.73
	4.45*E* − 01	4409.71	16.03	0.36
	8.90*E* − 01	9346.45	42.18	0.45

**Table 3 tab3:** Accuracy results.

Placebo (%)	Placebo (mg)	Bacopaside II (mL)	Bacopaside II (mg)	(%w/w)
0	0	1	0.223	100.00
80	16	1	0.223	104.97
100	20	1	0.223	102.76
120	24	1	0.223	100.51

**Table 4:** Extraction efficiency results.

Extraction number	%w/w bacosides	% recovered
1	9.235	97.7
2	0.213	2.30
3	0.000 (nd)	0.00

**Table 5:** Peak areas and retention times of replicate injections of standard solution at 0.089 mg/mL.

Replicate injection number	Peak area	Retention time (min)
1.00	936.26	27.882
2.00	929.30	27.809
3.00	936.67	27.886
4.00	945.46	27.875
5.00	925.90	28.009
6.00	936.10	27.935

Mean	**934.947**	**27.889**

STDEV	**6.783**	**0.067**

%RSD	**0.73**	**0.24**

**Table 6:** Results of method precision (1st operator) and intermediate precision (2nd operator).

Sample	Injection	Bacosides (%w/w)	
replicate	replicate	1st operator	2nd operator
1	1	1.02609	0.9489
	2	1.0181	0.9501
	3	1.0053	0.9494

2	1	1.03001	1.047
	2	1.05028	0.9872
	3	1.03453	0.97095

3	1	1.00957	0.97228
	2	1.02717	0.97656
	3	1.05503	0.9754

4	1	0.99785	0.938
	2	0.9765	0.97555
	3	0.9665	0.9839

5	1	1.09557	0.9748
	2	1.03263	1.0428
	3	1.04265	1.01844

6	1	0.9804	1.0184
	2	0.97473	0.9899
	3	0.99676	0.99117

%w/w (mean)		**1.018**	**0.984**

STDEV		0.031	0.024

% RSD		**3.00**	**2.46**

**Table 7:** Summary of validation results.

Test	Limits	Conclusions/results	
Specificity	No interfering peaks with that of the target.	Complies, however, bacoside peaks are coeluting	

Linearity (calibration coefficient)	The Y-intercept should not be more than ±2%.	0.38%	
	Linearity *R* ^2^ ≥ 0.990	*R* ^2^ = 0.9992	

Instrument precision	The % RSD of the retention time is ≤1.0%	0.24% RSD	
	The % RSD of the peak area is ≤10.0%	0.073% RSD	

Detection limit	S/N ≥ 3	0.00445 mg/mL	

Quantitation limit	S/N ≥ 10	0.0089 mg/mL	

Method precision	The mean result at method working strength is within the specification	Pass (1.02 %w/w)	
	The % RSD is ≤10.0%	3.0% RSD	

Intermediate precision	The mean result at method working strength is within the specification	Pass (1.02 %w/w)	
	The % RSD is ≤10.0%	2.5% RSD	

Extraction efficiency	>95% recovery from first extract	97.7% of total recovered	

Accuracy/recovery	At concentrations ranging from 80 to 120% of nominal stated content, the recovery Bacopaside II is 90.0% to 110.0%	Sample	%
		80	100.51
		100	102.76
		120	104.97

Range	Precision, accuracy, and linearity must meet their criteria from LOQ% to 150% of the label claim	0.00445–0.89 mg/mL	
